# Music as a perioperative, non-pharmacological intervention in veterinary medicine. Establishing a feasible framework for music implementation and future perspectives with a focus on the perioperative period of dogs and cats

**DOI:** 10.3389/fvets.2025.1672783

**Published:** 2025-10-14

**Authors:** Stefanos G. Georgiou, Apostolos D. Galatos

**Affiliations:** Clinic of Surgery, Faculty of Veterinary Science, School of Health Sciences, University of Thessaly, Karditsa, Greece

**Keywords:** dog, cat, perioperative period, anesthesia, analgesia, music

## Abstract

Research in human medicine has provided sufficient evidence to support music’s incorporation into the perioperative period, suggesting it as a significant non-pharmacological adjunct in terms of a multimodal approach, which should be available to all surgical patients. The literature regarding music’s effect on the perioperative period of dogs and cats is far more limited compared to that of humans, albeit quite promising. In order to design an individualized and potentially successful music intervention in dogs and cats, a stepwise approach is proposed taking into consideration the recommended components, such as music type (genre, tempo, pitch, instrumentation), volume, method of music delivery, duration and timing of the music intervention, frequency of music presentation and the effect of previous music experience. This review aims to provide directions to standardize perioperative music intervention protocols in dogs and cats, incorporate them into clinical practice and propose future perspectives, based on the existing literature evidence both in humans and companion animals.

## 1 Introduction

Research in human medicine is considered sufficient to provide enough evidence regarding music treatment during the perioperative period. Music-based interventions before, during or after surgical operations, under sedation or general anesthesia can be considered an effective, safe, inexpensive, and minimally invasive, non-pharmacological adjunctive therapy to alleviate procedural and postoperative pain, minimize perioperative anxiety, reduce sedative, anesthetic and analgesic requirements, ameliorate stress response to surgery or promote hemodynamic stability and should be available to all surgical patients (1–12). Music interventions have been applied to a wide variety of surgical settings ranging from minor endoscopic procedures to more invasive procedures such as orthopedic, abdominal, cardiothoracic and transplantation surgery, or even major surgery requiring intensive care stay, thus varying in terms of severity and patient health status (1, 2, 4, 6–9, 11). Although many studies have been conducted in human patients, it seems that the methodological approaches are characterized by significant variability, and systematic reviews and meta-analyses report high heterogeneity of the included studies, when music’s effect is being investigated (2, 4, 6–8, 13–16). That heterogeneity may be explained by methodological variations related to the type of music (3, 6, 14–16), the individual who chooses the music (2, 8, 14–16), the timing of the music intervention (2, 6–8, 14), the duration of the intervention (2, 6, 13, 16), the type of music delivery (8, 14), the type of surgery (2–4, 6, 8, 14), the method of anesthesia (4), the type of control condition (2, 4, 5, 16), or the theoretical framework underlying the effectiveness of music (14).

The literature regarding music’s effect on the perioperative period of dogs and cats is far more limited compared to that of humans, albeit quite promising. Only six published studies investigated the potential effect of music interventions during the perioperative period (17–22), but a larger number of studies have evaluated music’s effect on the welfare of dogs and cats. Existing evidence of a favorable response in the perioperative setting is limited to healthy patients (17, 18, 20–22), undergoing either elective ovariohysterectomy (17, 18) and minor skin surgery (22) or not submitted to a surgical stimulus at all (20), with only one study evaluating music’s effect postoperatively after more invasive procedures (21). The observed favorable responses that were reported under these conditions were significantly lower intraoperative values of heart rate (HR), respiratory rate (RR) and systolic blood pressure (SBP) (17, 18), anesthetic- (20, 22) and analgesic-sparing properties (21, 22), increased levels of sedation preoperatively (20) and lower postoperative anxiety (21). These findings along with the proposed music’s ability to modulate behavioral and physiological arousal in dogs and cats when used as an environmental enrichment adjunct seem very interesting, although inconsistencies between studies may limit the replication and generalization of these results. A recent critical review (23) reported a list of methodological issues which contribute to the variation in how music affects animal well-being, which seems to have a lot in common with the reasons for the reported variability in human studies. Such problems, among others, are the lack of control sounds, the lack of the underlying reason for the selection of a musical stimulus or the mechanism behind its selection, the absence of adequate information about the selected musical stimuli or about the delivery of music, and the absence of clearly stated study outcomes. More specifically, the current body of research on the perioperative application of music in dogs and cats is limited by several methodological issues that could reduce the reliability and clinical applicability of findings, as being acknowledged by most authors. Most studies report small sample sizes, often fewer than 20 animals per group (17–19, 21), while another limitation is the lack of proper blinding, especially when assessing subjective measures such as behavioral responses or depth of sedation, level of anxiety and pain scores (19–21). There is also heterogeneity in the design of music interventions, including variation in the type of music (species-specific music vs. classical, pop or heavy metal music), volume, duration and timing of exposure (17–22).

In order to incorporate music into dogs’ and cats’ surgical settings, some features of the music intervention need to be defined. Parameters such as the type of music (genre, characteristics of music stimulus, volume), the timing of the intervention (solely preoperatively, intraoperatively or postoperatively, or on multiple perioperative timepoints), the type of the surgical intervention, the individual who selects the music, the duration of the music intervention, the participant’s history and listening habits, and others seem to be important components to be considered when designing a perioperative music therapy intervention, according to systematic reviews and reported guidelines in human patients (2–4, 6–8, 14). Among these, a recent review that aimed to provide guidelines for musical selection and employment in surgical interventions proposed that a music intervention should follow a theoretical framework to create an effect or to justify its use (14). These parameters seem to be important for music interventions in dogs and cats, as well. However, we cannot make clear clinical recommendations about the essential features of music intervention in dogs and cats relying exclusively on veterinary literature, since only a small number of published studies are available, not to mention that the feline body of research is even more limited compared to the canine literature. So, it is preferable to outline the major components that should be considered in order to provide music during the perioperative period in dogs and cats, in terms of a multimodal anesthetic approach, by combining the already reported essential parameters in both human and veterinary studies, in order to propose some fundamental directions, until further research is conducted.

The current review aims to delineate the framework for music interventions during the perioperative period in dogs and cats, as a non-pharmacological adjunct in terms of a multimodal approach. It provides directional guidance for music implementation in a clinical setting, according to the existing evidence for music effects on anxiety, anesthesia and analgesia both in humans and companion animals, and proposes the potential components of a successful music intervention during the perioperative period in dogs and cats. Finally, future directions for research in the current field, regarding dogs’ and cats’ perioperative period, are proposed.

## 2 Recommendations for clinical application of music therapy during the perioperative period in dogs and cats. A stepwise design of the music intervention

### 2.1 Components of a music intervention

#### 2.1.1 General considerations

**Figure 1:**
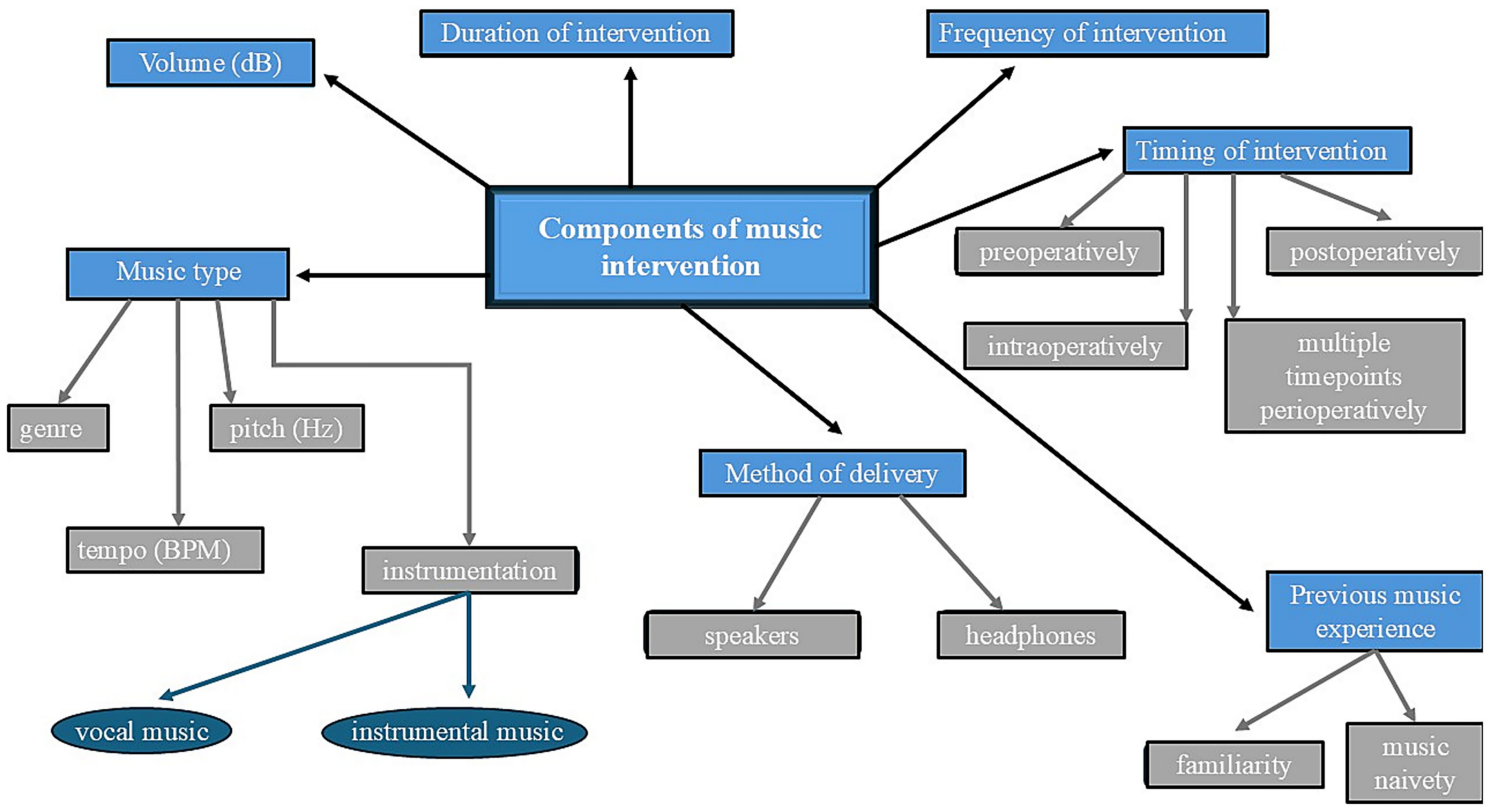
Parameters that should be considered when designing a perioperative music intervention on dogs and cats.

Veterinary clinics have been considered environments that can be stressful and challenging for dogs and cats, leading to high levels of stress, and negatively affecting their welfare (24–29), with those consequences potentially being reflected to the perioperative period, as well. Therefore, it seems mandatory to keep animals in a quiet area, rather than a loud and perhaps crowded environment once the premedication has been administered, as that is beneficial to a full sedation response (30). When trying to incorporate music therapy in a multimodal approach for the perioperative period of dogs and cats, that quiet area could be enriched with a properly provided acoustic stimulus, according to the recommendations analyzed below. The parameters that should be considered when designing a perioperative music intervention in dogs and cats are summarized in Figure 1.

#### 2.1.2 Music type (genre, tempo, pitch, type of instrumentation)

##### 2.1.2.1 Humans

Music type in humans is mostly described in terms of genre or other subjective characteristics such as “relaxing,” “soft” or “soothing,” and so, studies did not consistently use the same type of music. Apart from musical stimuli, other auditory stimuli have been used as well, like nature sounds, white noise, or therapeutic suggestions. However, it must be noted that nature sounds have not consistently shown the same analgesic effect as self-selected music (31), while intraoperative therapeutic suggestions had no effect either on patient recovery (9). The systematic review and meta-analysis of Fu et al. (9), which included 53 RCTs (randomized controlled trials) with 4,200 patients, found that intraoperative music of different genres (classical, jazz, new-age, piano music and others) significantly reduced postoperative pain and opioid requirements. However, a recent RCT, including patients subjected to esophageal and gastric cancer surgery under general anesthesia, found no effect of intraoperative classical instrumental music on postoperative pain, perioperative medication requirements or patient outcome (32). The observation that music’s beneficial effect may not be attributed exclusively to a specific type of music has been demonstrated both in settings related to the perioperative period (4) or not (31, 33). A meta-analysis evaluating the effect of music on perioperative anxiety and pain in patients undergoing invasive surgical procedures reported that the analgesic effect does not seem to depend on a specific type of music (4). The fact that the specific music genre is not of great importance for the observed analgesic effect has been demonstrated both in an acute pain setting (31) and in chronic pain settings (33). However, chaotic music such as hip-hop or heavy metal is not considered beneficial to humans (34). It has been proposed though that music features like tempo, rhythm, melody, and volume should be carefully considered in order to maximize the effect of a music therapy intervention (35).

Existing evidence suggests that relaxing music is more efficient than stimulating music for pain relief (35, 36), and the music-induced arousal effect is predominantly related to the tempo (37, 38). More specifically, listening to music with a fast tempo induces an increase in sympathetic nerve activity leading to arousal, while slower rhythms contribute to relaxation (37, 38). It is thought that music with a tempo of 60–80 BPM (beats per minute) would be ideal to promote a decrease in sympathetic activity and thus stress (14, 39). That particular tempo range, mimicking the HR at rest, has been hypothesized that facilitates the relaxation response (14, 34, 39), and instrumental relaxing 60–80 BPM music reduced preoperative anxiety to a greater extent compared to midazolam in 372 patients in the preoperative period (40).

Other features such as dynamics, pitch range, and texture were infrequently described in the studies included in a systematic review evaluating music’s effect on the perioperative period, despite evidence showing that they can impact a patient’s health (14). Dynamic and textural crescendos have been considered to cause increases in HR and respiration, while high pitches are frequently considered aversive, evoking anxiety and muscle tension, thus suggesting that these elements should be carefully considered (14). In the same context, variation in dynamic amplitude may cause arousal during music exposure (40), and music with abruptly shifting tones has been related to worsening symptoms in certain chronic pain conditions (35). Regarding vocal or instrumental music, a meta-analysis of 55 studies reported reductions in opioid requirements when human patients were exposed to either vocal or instrumental music during the perioperative period both at 24 and 72 h after surgery (8). However, the reported effects could be related to a larger extent to the instrumental music interventions rather than the vocal ones, as the majority of the included studies (45 studies, 82%) used non-lyrical or instrumental music interventions. Anyway, it has been proposed that music containing lyrics should probably be avoided as it may be distracting and activating (40). Finally, considering the instrumentation, melodies from harps, cello, and strings, either composed or arranged by music therapists, can be considered relaxant and can be applied in ICU (intensive care unit) patients (34).

##### 2.1.2.2 Dogs and cats

Results from veterinary studies have shown that music auditory enrichment may have a positive outcome compared to no music conditions in dogs (20–22, 41–47) and cats (17, 18, 48–50), in different dog and cat populations and in different environments. However, other studies found no apparent effect of music on dogs (29, 51, 52). From those reporting a positive effect, a potential music type-dependent beneficial effect has been assumed for dogs (41, 42) and cats (17, 18); however, others propose that the effect is not genre-specific on dogs (45) and cats (50).

Classical music has been considered to influence behavioral and physiological parameters, such as HRV (heart rate variability), vocalization level and time spent resting (53). Dogs exhibit calmer and more relaxed behaviors when exposed to classical music compared to heavy metal, rock, pop music, psychoacoustically designed dog music, human conversations, or no music in potentially stressful environments such as boarding kennels, rescue shelters and veterinary clinics (41–46). However, other studies reported no significant effect of classical music (29, 47, 51), or dogs’ preference for other music genres (45). Bowman et al. (45) found that other music genres such as soft rock, Motown, pop and reggae induced behavior changes indicative of reduced stress apart from classical music, compared to silent conditions. Another study observed that an audiobook exhibited the most beneficial effects compared to all other treatments (pop music, silence), even compared to Beethoven’s music (44). However, exposure to an audiobook did not benefit pet dogs separated from their owners in another study (46). Furthermore, no clear benefit was suggested by bespoke music (designed to entrain physiological parameters of dogs, composed by a professional music producer) (29), or psychoacoustically designed dog music (Through a Dog’s Ear) when compared to a random selection of classical music (42, 44, 51, 52). Surprisingly, Koster et al. (52) reported that dog relaxation music had an excitatory rather than a calming effect. Anyway, playing soothing background classical music and avoiding hard rock or heavy metal music has been recommended by guidelines for pet-friendly veterinary practice, to maximize the environmental comfort of dogs (30), exhibiting, at best, a moderate calming effect (46). In cats, the existing literature seems to support the implementation of both music specifically designed for them (48–50) and classical music (50) for stress reduction, compared to silence, in different environments. Playing calming classical music or cat-specific music has been proposed to create a more relaxed atmosphere by recent guidelines for a cat-friendly manipulation of the veterinary clinic environment (54).

Regarding the effect of music implementation during the perioperative period in dogs and cats, published studies used either classical music pieces of different composers (17, 18, 20, 22) or music specifically designed for dogs (19, 21). Apart from the study of Albright et al. (19), who found no benefit of music designed for dogs on the level of dexmedetomidine-induced sedation, the rest of the studies reported variable favorable effects of classical music implementation on the perioperative period of both dogs (20–22) and cats (17, 18) compared to either no music silent scenarios (17, 18, 20–22) or other music genres (17, 18). Classical music pieces of Mozart and Chopin contributed to an increase in the sedation level and an approximately 20% reduction in propofol requirements for intubation when implemented preoperatively in laboratory Beagles compared to a silent control group (20), and, when the same excerpts were applied intraoperatively under a light anesthetic plane on a similar dog population, an anesthetic and analgesic sparing effect was demonstrated compared to the group not exposed to music (22). Apart from human classical music, auditory stimulation with classical music designed for dogs, as part of an integrative environmental approach, exhibited a beneficial effect on dogs’ postoperative period after hemilaminectomy (21). Dogs recovering in an environmentally enriched room received less rescue analgesia and consumed more food compared to dogs recovering in the standard environment of an intensive care room. Classical music appeared to exhibit desirable responses in cats under general anesthesia for elective ovariohysterectomy, not only compared to a silent control condition but also compared to pop and heavy metal music (17, 18). Most cats exhibited lower mean RR, PD (pupil dilation), HR and SBP values when exposed to classical music, intermediate values to pop music and higher values to heavy metal music.

Apart from the genre of music, other features such as tempo, pitch range or instrumentation have been investigated for their potential impact on a successful music intervention in dogs and cats. Slow-tempo classical music exhibited a calming effect on kenneled dogs (41–43). Kogan et al. (42) mention that the selected classical musical pieces averaged around 121 BPM and that the specifically designed dog music had an average of 95 BPM. However, classical music exhibited more beneficial effects compared to dog-designed music in that study, although classical pieces had a slightly higher tempo average. In the aggregate, although dog music is designed as slow-tempo music (50–60 BPM) it was not proven to be superior to other slow-tempo classical music selections, even of higher tempo (42, 44, 51). Further evidence supporting that slow tempo is not the only feature of music to consider when designing a music intervention is provided by Amaya et al. (55) who did not find significant differences in the activity of dogs when exposed to slow-tempo music (of 70 BPM or fewer) compared to a 30% faster tempo. Something similar was observed in all existing cat studies, where cat-specific music with an extremely high purring- or suckling-related tempo (>250 BPM) resulted in behaviors indicative of less stress compared not only to silence (48–50) but also to classical music, with a much lower tempo averaged around 56–66 BPM (48, 49).

Studies related to the perioperative period did not reach definitive conclusions regarding the optimal tempo of music interventions in dogs and cats, as well. Slow-tempo Chopin and Mozart excerpts (lento sostenuto, andante of approximately 50–100 BPM) were more advantageous compared to no music preoperatively and intraoperatively (20, 22). Low-tempo classical music specifically designed for dogs had a positive impact on the postoperative period (21), but another study using the same type of music intervention found no effect on the level of sedation preoperatively (19). Although the respective music, marketed to produce a calming effect in dogs, is a slow-tempo collection of approximately 50–60 BPM, these conflicting results support the hypothesis that standardizing only the tempo of the music intervention may not be enough. Furthermore, Mira et al. (17, 18) did not contribute further to that context as they did not mention the exact tempo of any of the 3 parts of the classical music piece that was used in their studies in cats.

In addition to the music genre or tempo, tonality or even instrumentation are probably important features for a music intervention (55). Bowman et al. (43) found that dogs appeared more relaxed, displaying signs of reduced physiological and psychological stress according to HRV and behavioral data when exposed to low-pitch, slow-tempo classical music; however, a more recent study (55), comparing pitch- and tempo-modified musical pieces which were previously proved to be beneficial in dogs (27) found that differences in behavior were only found when compared to low-pitch music, although slow-tempo music was expecting to produce the most relaxing effects (55). Amaya et al. (55) found that low-pitched tracks could be perceived as disturbing by dogs, increasing their alertness as evidenced by “arousal” tail movements. Although we do not have enough data for pitch characteristics of music interventions in dogs, the appropriate music for cats seems to be more detailed regarding the pitch range. Cat-specific music compositions, which appeared pleasant for an agitated cat, were designed to contain purrs and suckling sounds at high frequencies similar to the cat vocal range (averaged 1.43 kHz), which is 2 octaves higher than the human vocal range (48–50). Regarding the instrumentation of the music interventions, piano-based simple instrumentation or string excerpts have been used for dogs (27, 42, 44, 51, 52, 55) and cats (48–50). That pattern has been also observed during the perioperative period with the majority of the interventions being piano-based (19–22) or using string orchestra music pieces (17, 18). However, no clear conclusions can be drawn, as other studies reported beneficial effects (17, 18, 20–22, 27, 42, 55), while others observed no effect from that type of instrumentation (19, 44, 51, 52).

##### 2.1.2.3 Recommendations for the music type

Researchers contend against overgeneralization of results solely in terms of genre, being a subjective human definition and a highly heterogeneous category (23). However, the perception that classical music is beneficial persists. Considering the existing literature data, classical music seems to be the best choice for the dogs’ perioperative period compared to other genres, while cats have demonstrated desirable responses when exposed to both classical music and music specifically designed for them. However, the music genre is not the only music feature that plays a role in a successful music intervention. Slow tempos, lacking abrupt dynamic alterations and simple instrumentations, potentially piano-based, have been considered to be the most beneficial. Finally, species-specific differences in the auditory range between humans, dogs and cats should be carefully considered, as the direct translation from human to veterinary medicine may not be applicable (humans perceive frequencies between 20 Hz and 20 kHz, while dogs and cats exhibit a wider hearing range of approximately 65 Hz to 45 kHz and 48 Hz to 85 kHz, respectively) (56, 57). The pitch range should conform specifically to that of the targeted species (dog or cat), as low-pitched excerpts should be avoided in dogs, while cats seem to prefer higher frequencies, compared to the human vocal range. Anyway, it is recommended to avoid abruptly shifting tones and overstimulation of the patient.

#### 2.1.3 Volume (intensity)

##### 2.1.3.1 Humans

Volume is one of the parameters that should be considered regarding music characteristics, including tempo and melody (35). However, a systematic review evaluating music interventions perioperatively found that the volume of music was generally under-reported, as listed only in 34.3% of the included studies (14), although it has been suggested that sound levels should be carefully controlled to avoid auditory adverse effects (35). The Occupational Safety and Health Administration (OSHA) reports that sustained sounds above 70 dB (decibel) can cause permanent hearing damage in humans (3). Most RCTs reported that patients adjusted the music volume according to their preferences to a comfortable level regardless of the timing of the music intervention (40, 58–62), while in others where the sound volume was preset, it ranged from 45 to 70 dB (63–65). According to a systematic review, the optimal music intervention sound volume that should be applied is unclear (2); however, later reviews proposed sound levels of 40–70 dB during the perioperative period (14), or a maximum volume of 68 dB specifically for patients under general anesthesia, to prevent the risk of permanent hearing damage (3). Anyway, the most recent recommendations for humans propose to restrict music volume and adhere to the noise and hearing loss guidelines in order to prevent hearing damage, to allow patients to choose a suitable music volume for them, or to use devices with limits on volume output (3, 8, 14).

##### 2.1.3.2 Dogs and cats

The volume of the music intervention seems to be of importance in dogs and cats, as well, considering that noise stimulation has been reported to cause acute increases in cortisol production consistent with a stress response in dogs (66, 67), while it has been proposed that keeping noise levels below 60 dB is preferable when considering a pet-friendly environment (30). In most studies in dogs, the volume of the auditory intervention, when implemented as an environmental enrichment tool or as an adjunct to the perioperative period, ranged from 43 to 73 dB (19, 20, 22, 27, 44, 46, 51, 52, 55), but some studies did not mention the exact volume of the auditory stimulation (21, 29, 41–43, 45). However, they pointed out that the intervention was either low-volume (21), or that the dynamic range of the music was narrow, lacking sudden volume peaks (29), or that the volume was stable throughout the study (43, 45), indicating that all researchers considered the volume of the intervention important. The same volume range pattern was observed in studies in cats where sound levels of 60–70 (48, 50), or <80 dB (17, 18) were reported. When higher volume levels (80–85 dB) were applied in dogs, the quality of dexmedetomidine-induced sedation was negatively impacted compared to lower volume conditions (19). More specifically, the dogs in that study were more deeply sedated when exposed to background noise of 40–45 dB compared to human voices at 80–85 dB. However, exposure to music of 45–50 dB did not contribute to a more profound sedation, compared to the 40–45 dB background noise condition. On the other hand, exposure to music at 50–65 dB resulted in an increased sedation level and a subsequent reduction in propofol dose requirements for intubation compared to a silent condition, in another study in dogs (20). The conflicting results between the studies of Albright et al. (19) and Georgiou et al. (20) concerning the music’s effect may be attributed to differences in methodology, such as the sample size (10 dogs versus 20 dogs), the type of the premedication protocol (dexmedetomidine versus acepromazine and butorphanol), or the duration of the exposure to the music (20 min versus 90 min). Anyway, music interventions of low intensity have proved beneficial during the perioperative period in both dogs and cats, so far; low-volume or up to 65 dB music stimulation was effective both intraoperatively and postoperatively in dogs (21, 22) and volumes of <80 dB were related to desirable manifestations in cats under general anesthesia (17, 18).

##### 2.1.3.3 Recommendations for the music volume

Animals begin to show symptoms indicative of stress when ambient sounds approach 85 dB (30, 68), sound levels that have been reported in veterinary hospitals (19). According to the existing literature on dogs and cats, music interventions should be low volume, not exceeding 65 dB. Furthermore, the volume should be stable throughout the intervention without sudden intensity changes which could overstimulate or stress the animal.

#### 2.1.4 Method of music delivery

##### 2.1.4.1 Humans

Headphones, music pillows, or background sound systems have been proposed in humans as methods of music delivery during the perioperative period (2). A more recent systematic review assessing the effect of perioperative music reported that the majority of the included studies (75%) used headphones for music delivery (8). Offering music via headphones during operations under regional anesthesia, with or without sedation, is already common practice (10), and Flanagan and Kerin (3) proposed that the use of volume-protective headphones can benefit patients exposed to music under general anesthesia. The use of noise-canceling headphones ensures the patient’s personalized delivery and masks the potentially disturbing operating room sounds (3, 8, 34). However, there is no clear advantage of one method of delivery compared to the others (2).

##### 2.1.4.2 Dogs and cats

When music interventions were used to relieve stress and anxiety in dogs and cats in different stressful settings such as boarding kennels, rescue shelters and veterinary clinics, either as a means of environmental enrichment or as an adjunct during the perioperative period, speakers and headphones were the methods of music delivery reported by all studies. Speakers were the predominant method of music delivery in dogs (19, 20, 27, 29, 42–47, 51, 52, 55) and cats (48, 50), while headphones covering the whole ear were used when dogs and cats were exposed to music under anesthesia (17, 18, 22). Only two studies did not mention the exact method of music delivery: one study in dogs exposed to music during the postoperative period (21), and one study in cats when exposed to music in the examination room of a veterinary teaching hospital (49).

##### 2.1.4.3 Recommendations for the method of music delivery

When music is incorporated into a multimodal approach during the perioperative period in dogs and cats, speakers or headphones covering the whole animal’s ear could be used. Loudspeakers should be used during the preoperative or postoperative period, while headphones should be adjusted properly during the intraoperative period. Apart from direct music delivery, the placement of headphones under anesthesia may contribute to the blocking of the operating room sounds or could help in terms of blinding in a research setting.

#### 2.1.5 Who chooses the music and previous music experience (e.g., familiar music)

##### 2.1.5.1 Humans

A common point of contention in human studies is whether patient- or researcher-chosen music is more effective. The evidence points to patient-preferred or self-selected music, as being the most effective both for acute (2, 4, 7, 11, 69–71) and chronic pain management (33, 35, 72). However, some of the aforementioned studies were conducted in healthy individuals subjected to an acute cold pressor nociceptive stimulus (71), or chronic pain patients (33, 35), and not in surgical patients. Regarding the perioperative period, two systematic reviews showed that music interventions utilizing patient-selected music were more effective in reducing postoperative pain levels (3, 11). Similarly, another systematic review reported that the largest beneficial effect on both anxiety and pain was seen when patients selected music from a playlist provided, according to their preferences, and proposes that individual music preferences should be considered when designing the music therapy plan (4). However, the results of three other systematic reviews were not so clear, as the choice of music made little difference to outcomes (2, 5, 7). More specifically, when surgical patients were allowed to choose their preferred music, either as a personal choice or from a given playlist, insignificant pain reduction was observed compared to patients who did not choose the music (2, 7). However, those authors still recommend that patients should be encouraged to choose the type of music they would like to hear (2). Listening to familiar music has been proposed to evoke a feeling of control, patient empowerment and pleasure, and, besides, to have a positive effect on physiological responses (3, 34, 73). As a pleasant experience, familiar music listening has been associated with increased activity in limbic, paralimbic and reward structures of the brain and a concomitant dopamine and endogenous opioid release, contributing to analgesic and relaxant effects (15, 33, 34, 73).

##### 2.1.5.2 Dogs and cats

Although it seems that self-selected music is more effective in humans compared to researcher-selected music, this could not be directly applied to veterinary medicine, as dogs and cats cannot choose the music intervention on their own, according to their individual preferences. Nevertheless, that component of music interventions could be assigned to the owner, considering the potential pet’s familiarity with certain music compositions or music types, or, if there is no such previous exposure, the type of music intervention is decided by the veterinary surgeon.

Most of the conducted studies mention no information about previous exposure of the included dogs to music, as part of their daily routine (27, 29, 41–45, 51, 55). However, some of these authors indeed recognize that dogs may have individual preferences for certain songs or music types, which may be related to their background experiences or previous exposure to music (29, 43, 45). Only a recent study investigating the effect of different auditory stimuli on dogs’ reactions to owner separation refers to the auditory stimulation background of the included dogs, reporting that they were routinely exposed to a wide variety of acoustic stimuli such as radio or TV, as part of their daily lives (46). However, a study limitation, as reported by the authors, was the unknown degree to which the dogs included were familiar with the types of employed auditory stimulation, thus recognizing that such a detail may play a significant role when designing a music therapy intervention. Based on that assumption, a more recent study aimed to evaluate the role of acoustic stimuli familiarity in specific behavioral or emotional responses in dogs (47). In that study no significant behavioral responses indicative of preference or aversion were observed when music-naive dogs were exposed to classical music; however, dogs that were routinely exposed to classical music at home exhibited more relaxed behaviors. Dogs familiarized with classical music spent twice as much time in the sound zone compared to those not used to classical music listening, suggesting familiarity-induced preference and familiarity-related emotional responses (47). So, some dogs may not like music at all or have a variety of positive or negative associations with particular music types related to previous experiences, influencing their responses (47, 52, 74). However, another factor that could result in a negative impact may be the exposure to a musical stimulus in an unfamiliar environment (29). This could be a potential explanation for the results of the study of Koster et al. (52), which demonstrated that when music specifically designed for dogs was applied to naive dogs, an excitatory rather than a calming effect was observed. This could be either attributed to an aversion toward the specific musical stimulus or to the potentially unfamiliar or stressful environment of music exposure. In that context, it has been proposed that if any adverse effects are observed due to specific songs or the music genre, then the music collection should be changed (42). Furthermore, to maximize the effect of the music intervention, music should be firstly applied to a familiar, or if not possible, to a more relaxing environment, especially before exposure to a stressful event. King et al. (29) recommended that music could be played immediately prior or during activities that promote pleasure and relaxation to the dog (feeding, playing, petting) in a secure environment, such as home, hypothesizing that a conditioned stimulus like music could produce pleasure, relaxation and calmness to a different environment, as well. So, apart from the familiarity concerning music interventions, dogs should be exposed to a familiar music piece in a familiar, or relaxing environment, with these components being interconnected. In cats, although all studies investigating music’s effect demonstrated a preference for feline-specific music or classical music (48–50), the authors of the respective studies did not mention any previous exposure to music, as part of their daily routine. Regarding familiarity, only Snowdon et al. (48) reported that the selected music pieces were presented only once during the experiment, so they were novel to the cats; thus, recognizing that potential prior exposure could be of importance or could alter the results.

Although studies investigating the effect of music interventions in dogs and cats during the perioperative period are far more limited than those evaluating music’s effect as an environmental enrichment strategy, some of the aforementioned conclusions could be adapted to the perioperative period, as well. Of the six studies that have been conducted on dogs and cats during the perioperative period, three make no mention of the previous exposure to music as part of their daily routine (17, 18, 21). The rest of the studies, all conducted in dogs, mention that they included only music-naive dogs (19, 20, 22). Albright et al. (19) mention that the dogs used in the study were purposefully bred for research and had limited or no exposure to music. In order to provide a potential explanation for the absence of any beneficial effect of music, the authors further report that including household dogs with prior music exposure may have resulted in different conclusions regarding music’s effect on behavior and sedation. However, the authors of the particular study tried to acclimatize dogs to the auditory stimulus by playing music and voice recordings, similar to those used in the experimental phase, 2 days before the experiment, recognizing that a novel auditory stimulus applied to dogs in a novel environment could result in unwanted effects. Regarding the other two studies which also included music-naive, purpose-bred laboratory Beagles, no music-acclimation period was reported (20, 22). However, in the study of Georgiou et al. (20) preoperative music was applied to dogs for 45 min before premedication, potentially serving as a music-acclimation period, while in the more recent study of the same research team (22), music was employed intraoperatively. Anyway, music exposure resulted in favorable outcomes, in both studies, although dogs were not familiar with music listening.

Overall, the existing literature underscores that considering the music background of the veterinary patient, receiving the related information from the owner when designing the music intervention approach could be of importance. It seems that the potential animal’s familiarity with music should be considered, along with the other main components of a music intervention, when creating a music therapy plan.

##### 2.1.5.3 Recommendations for previous music experience

Patient-chosen music therapy offers the advantage of patient empowerment and patient-centered care in humans (3); however, music cannot be patient-chosen in veterinary medicine. The decision regarding the type of music intervention is assigned to the veterinary professional, except for the case where a thorough history-taking reveals that the animal is familiarized with certain music compositions or music types. In that case, the respective type of music should be considered, especially if it is associated with relaxing or pleasant situations. Familiarity can help create a sense of comfort and potentially reduce anxiety in a stressful situation such as the perioperative period and in a stressful environment like the veterinary clinic.

If no information about previous music listening exists, then the designing of the music therapy plan should be based on the other proposed parameters. In that case, the animal should be firstly acclimatized to the novel environment of the preparation area and afterwards the preoperative music intervention should begin, probably in the company of a familiar person, such as the owner. If the animal exhibits aversive behavior, then the playlist should be changed. If the animal remains calm and relaxed, then the anesthetist should proceed with the administration of the premedication and wait for its maximum effect to be achieved.

#### 2.1.6 Duration of music intervention

##### 2.1.6.1 Humans

Although it has been proposed that documenting the duration of a music intervention is of great importance in order to evaluate its effect (14), systematic reviews and meta-analyses report that it is difficult to draw a definite clinical recommendation regarding the optimal duration of a perioperative music intervention (2, 4, 8). However, literature data regarding the effect of music interventions on a variety of study outcomes in different populations, such as surgical, ICU, or chronic pain patients, report a minimum of 15-min interventions and a maximum of 120 min (2, 8, 13, 14, 31, 34, 39, 70, 72). Systematic reviews of the effect of perioperative music interventions found that relatively short music exposure can be beneficial; Hole et al. (2) reported that the most common approach was to employ music for 20–60 min, Williams & Hine (14) mention that the interventions were predominantly 30-min long, while Fu et al. (8) in their review including 55 studies (4,968 patients) found that 75% of studies assessing opioid requirements exposed patients to a total of 120 min of perioperative music on average. A literature review aiming to develop a best-practice protocol for the perioperative use of music therapy, recommends that music interventions should have a duration of at least 15–30 min preoperatively and postoperatively (39). In that context, 30-min music interventions during the postoperative period in patients subjected to mechanical valve replacement surgery resulted in a decrease of both acute postoperative and chronic pain (72). Regarding the duration of music exposure in ICU patients (mainly postoperative patients), music interventions of 20–30 min were associated with a larger decrease in pain scores compared to those of less than 20 min (70), while music interventions of more than 60 min were associated with more pronounced reductions of serum cortisol levels compared to 30-min interventions (13).

##### 2.1.6.2 Dogs and cats

It seems that the duration of music exposure could be a valuable component of the music intervention in animals, as well. However, the existing evidence in veterinary literature is far too limited to draw a conclusion about the ideal duration of music intervention in dogs and cats, let alone regarding the perioperative period.

The methodology of the studies in dogs and cats seems to be inconsistent. Some studies used only one type of music throughout the music intervention (19–22, 44, 46, 47, 49, 52), while others used a variety of stimuli in the same session, either from the same origin, e.g., the same type of music (27, 29, 41, 43, 45, 50, 51), or from different music types and with different characteristics, e.g., variations according to music genre, tempo or instrumentation (17, 18, 42, 48, 55). Other studies used a single music track repeated on a loop for the duration of the testing period (20, 22, 46, 49), while others used a playlist of compositions (17–19, 21, 27, 29, 41–45, 47, 48, 50–52, 55). Finally, some studies incorporated a control, silent condition into the same music intervention period (17–19, 42, 48, 55), while others used the control condition at a separate testing period (20–22, 27, 29, 41, 43–47, 49–52).

Anyway, the duration of the music intervention when music was used as an environmental enrichment method or as a stress-relieving strategy ranged from 15 min to 6.5 h in dogs, while the respective duration of the music interventions in cats ranged from 3 min to 4 h. When the music interventions exceeded 2 h a favorable response related to reduction in arousal behaviors was demonstrated in most studies in dogs (27, 41–45), while a study found that a 15-min music intervention (classical music) before clinical examination during a veterinary visit had no apparent effect on dogs’ stress (29). In cats, all conducted studies demonstrated beneficial effects on stress levels, either when music was applied for 3 min in the home environment (48), or when applied for 10 min in a veterinary clinical setting during a clinical examination (49), or when music was played for 4 h, twice a day, during hospitalization (50). Some of the observed adverse effects in dogs were not exclusively attributed to the duration of the music exposure. For example, some studies reported that dogs exhibited signs of nervousness after exposure to heavy metal music (41, 42), while another study reported that dogs became refractory to the physiological and psychological effects of music when the same playlist was used repeatedly for 6.5 h per day for a total of 7 days (43). However, those adverse effects were attributed to other components of the music intervention, such as the music type or the frequency of the intervention and were not duration-dependent.

Regarding the perioperative period, music interventions in dogs ranged from 20 min to 8 h (19–22), depending on the phase of the perioperative period, while the two existing studies in cats used a 6-min intraoperative music stimulation in total, including three different types of music with a 2-min duration each (17, 18). Twenty minutes of music, while waiting for the peak dexmedetomidine’s effect, had no apparent effect on the achieved level of sedation in 10 dogs (19), while 90 min of classical music contributed to higher sedation scores and a 20% reduction in propofol requirements for tracheal intubation in 20 purpose-bred laboratory Beagles (20). Someone may conclude that longer than 20 min of music interventions can potentially be more effective compared to those lasting less than 20 min. However, the methodology of the last two studies is very different and their results cannot be comparable. These two studies differed from each other not only regarding the duration of the music intervention but also regarding the sample size, the type of the music intervention and the premedication protocol, among others. Beneficial effects were also demonstrated when music was applied during the intraoperative period either under a light anesthetic plane for approximately 90 min in dogs (22), or even under general anesthesia for as little as 6 min in cats (17, 18). Longer music interventions lasting approximately 8 h per day, were associated with improvement in postoperative pain and anxiety in dogs for the first 48 h after hemilaminectomy (21); however, the beneficial effects in the particular study cannot be exclusively attributed to the music or its duration, as multiple methods of environmental enrichment were evaluated simultaneously.

##### 2.1.6.3 Recommendations for the duration of music intervention

Although firm conclusions cannot be drawn, the optimal duration of a music intervention seems to be in accordance, more or less, with the directions applied to humans. When incorporating a music intervention into the perioperative period of dogs and cats, its duration should be preferably at least 20–60 min. Depending on the phase of the perioperative period, the intervention could be extended, using continuous play during the perioperative period. However, music should not be a permanent, non-stop, all-day auditory stimulus.

#### 2.1.7 Timing of music intervention

##### 2.1.7.1 Humans

Literature evidence proposes that music would be an effective supplementary tool throughout the perioperative experience, either before, during or after surgical procedures (2, 4, 5, 7–9, 39). A literature review trying to establish a consensus regarding perioperative music therapy recommends that music should be played throughout the entirety of the procedure or surgery (39). Music interventions preoperatively, intraoperatively, or postoperatively were found to reduce postoperative pain levels (2, 4, 7, 8), sedative (8) and analgesic requirements (2, 4, 8), anxiety levels (2, 4) and furthermore reduced treatment costs and minimized the risk of adverse effects (8).

Some systematic reviews found that the timing of music delivery made a minor difference to the study outcomes (2, 4). The systematic review of Hole et al. (2) found a greater effect on pain when music was played preoperatively compared to intraoperatively, and the smallest effect when delivered postoperatively, but the current sub-group analysis was of high heterogeneity. A similar pattern was observed both for postoperative analgesic requirements and anxiety levels, as analgesia use and anxiety levels were mostly reduced when music was played preoperatively, compared to intraoperatively or postoperatively (2). The same results regarding anxiety levels were reported by a subsequent systematic review and meta-analysis of 81 RCTs showing that the largest effect was observed when music was offered before surgery (4). However, a subgroup meta-analysis of the current study found different results on pain levels compared to the study of Hole et al. (2); Kuhlmann et al. (4) found that the largest effect on pain reduction was demonstrated when music was played postoperatively. Anyway, music-mediated postoperative pain and anxiety reduction was significant when music was applied before, during or after the surgical procedure (4).

A distinct reference should be made to the effectiveness of music during the intraoperative period when patients are under general anesthesia. Evidence exists for preserved auditory sensory perception under general anesthesia, as the primary auditory cortex remains receptive and reactive to auditory stimuli even during deep sedation (75, 76). A dose-dependent suppression of auditory function by anesthetics has been proposed for humans (75, 77–79) and this goes in parallel with the results of Krom et al. (80) whose findings demonstrate that anesthesia-induced loss of consciousness disrupts auditory responses beyond primary cortex while significant responses persist in the primary auditory cortex. Even when music was applied solely under general anesthesia a significant decrease in pain levels was demonstrated (2, 4); however, the intervention’s effect on pain was greater when patients were conscious, compared to when being under general anesthesia (2). The results of a recent systematic review and meta-analysis evaluating memory formation during general anesthesia and its effects on postoperative patient outcomes and recovery showed that intraoperative auditory stimuli can be perceived and processed during clinically adequate general anesthesia, leading to implicit memory formation without explicit awareness (9). This study reported a significant moderate to large beneficial effect of intraoperative music during general anesthesia on postoperative pain and opioid requirements for the first 24 h after surgery, when pain levels are considered to be the highest (9). Similar beneficial results of music therapy under general anesthesia were also demonstrated by a former systematic review (3); however, a recently published RCT including patients subjected to esophageal and gastric cancer surgery under general anesthesia, found no effect of intraoperative instrumental music on postoperative pain and perioperative medication requirements (32).

##### 2.1.7.2 Dogs and cats

The number of existing studies on dogs and cats is far too limited to draw conclusions regarding the optimal timing of the music intervention. Furthermore, the majority of the existing veterinary studies evaluated only single-phase music interventions; Albright et al. (19) and Georgiou et al. (20) applied music to dogs only in the preoperative period, Pennington et al. (21) used music auditory stimulation as part of an integrative environmental enrichment approach solely in the postoperative period in dogs after hemilaminectomy, while Mira et al. (17, 18) used music stimulation in cats only intraoperatively. So, no data is available regarding the potential effect of those single-phase music interventions on the forthcoming phases of the perioperative period. More specifically, there is no information about the further effect of preoperative music, in the case that the dogs in the study of Georgiou et al. (20) would undergo a surgical operation, considering the observed effect on sedation and propofol requirements. Furthermore, we have no information about the potential effect of intraoperative music, on the postoperative period in the dogs of the studies of Georgiou et al. (22), or in the cats of the studies of Mira et al. (17, 18). However, the studies of Mira et al. (17, 18) and the study of Georgiou et al. (22) provide indirect evidence that auditory sensory stimuli processing may persist under light plane anesthesia in dogs or under general anesthesia in cats.

Anyway, most of the studies where a music intervention was employed reported beneficial effects related to the phase of the perioperative period that music was applied (17, 18, 20–22), apart from the study of Albright et al. (19) where no effect of music was observed on the level of sedation.

##### 2.1.7.3 Recommendations for the timing of music intervention

If music is to be incorporated into a multimodal approach, it is proposed to be applied during the preoperative period, throughout the surgical procedure and during recovery, targeting a calming environment. Music should be offered to dogs and cats preoperatively to reduce stress, anxiety and excitement, maximize the premedication’s effect and promote a smooth induction process. It should be applied either while in the waiting area or preparation room or while waiting for the peak effect of the premedication. Music during the intraoperative period could contribute to stable anesthesia and physiological homeostasis maintenance. Postoperatively, music could promote calmness during recovery and reduce pain and stress. Enrichment of the recovery area with music may help animals recover more smoothly and reduce postoperative complications related to stress and anxiety due to the owner’s absence or due to the unfamiliar environment. Anyway, the timing of the intervention should be individualized, depending on the desired, anticipated effect.

#### 2.1.8 Frequency of music intervention

That component of a music intervention could not be totally relevant to the perioperative period, as the duration of the preoperative or the intraoperative periods is specific and directly related to the surgical procedure. That means that during the perioperative period music is typically applied once as a single intervention, apart from the postoperative period where music can be applied intermittently. However, the frequency of the music interventions could be of interest regarding the postoperative period, depending on the length of hospitalization.

##### 2.1.8.1 Humans

There is not enough data to support a firm conclusion about the frequency of presentation in humans, as most systematic reviews and meta-analyses were focused on the immediate postoperative period. The guidelines by Poulsen and Coto (39) recommend that music should be administered consistently at least twice daily during the postoperative period, to be most effective. In that context, music therapy decreased persistent post-surgical pain and improved the quality of patient’s life, when they listened to music once a day for 6 months after mechanical valve replacement surgery (72), while regarding ICU patients, intervention frequency ranged from 1 to 3 times per day and lasted up to 30 days (34).

##### 2.1.8.2 Dogs and cats

In the case of the perioperative period in veterinary patients, music interventions could be intermittently applied to the hospitalized dog or cat as part of a multimodal approach. Concerning the existing literature, when music was employed preoperatively (19, 20) and intraoperatively (17, 18, 22) in dogs and cats, it was offered only once. However, in the study of Pennington et al. (21), where music was incorporated into an integrative environmental enrichment approach during the postoperative period in dogs recovering from hemilaminectomy, music was offered for at least 8 h per day for the 48 h of hospitalization. No definite conclusions can be drawn about the optimal frequency of music presentation postoperatively solely from that study, but there have been reports of dogs’ habituation to the effects of music when they were exposed to the same playlist for 6.5 h per day for 7 days (43). More specifically, it was reported that the calming effects of music observed on the 1st day were not maintained until day 7 and were already lost by the 2nd day of exposure. However, this was more attributed to the fact that the same playlist was used repeatedly, than to the duration of the music exposure or the frequency of its presentation, as subsequent studies reported no evidence of habituation when a variety of different genres was provided for 6 h per day for 5 days (45), or when the playlist was provided with random order, for 3 h per day for five consecutive days (27). Anyway, no apparent evidence of habituation was reported in the study of Pennington et al. (21), when music specifically designed for dogs was offered for at least 8 h per day for 2 days.

##### 2.1.8.3 Recommendations for frequency of music intervention

The authors encourage the use of music interventions during the postoperative period. The playlist should be individualized, taking into consideration the components of a music intervention that are mentioned above. The tracks should be in random order in each intervention, but a short playlist or a single-track intervention repeatedly should be avoided. Finally, the sessions should be provided either once or twice a day.

## 3 Conclusion

**Figure 2:**
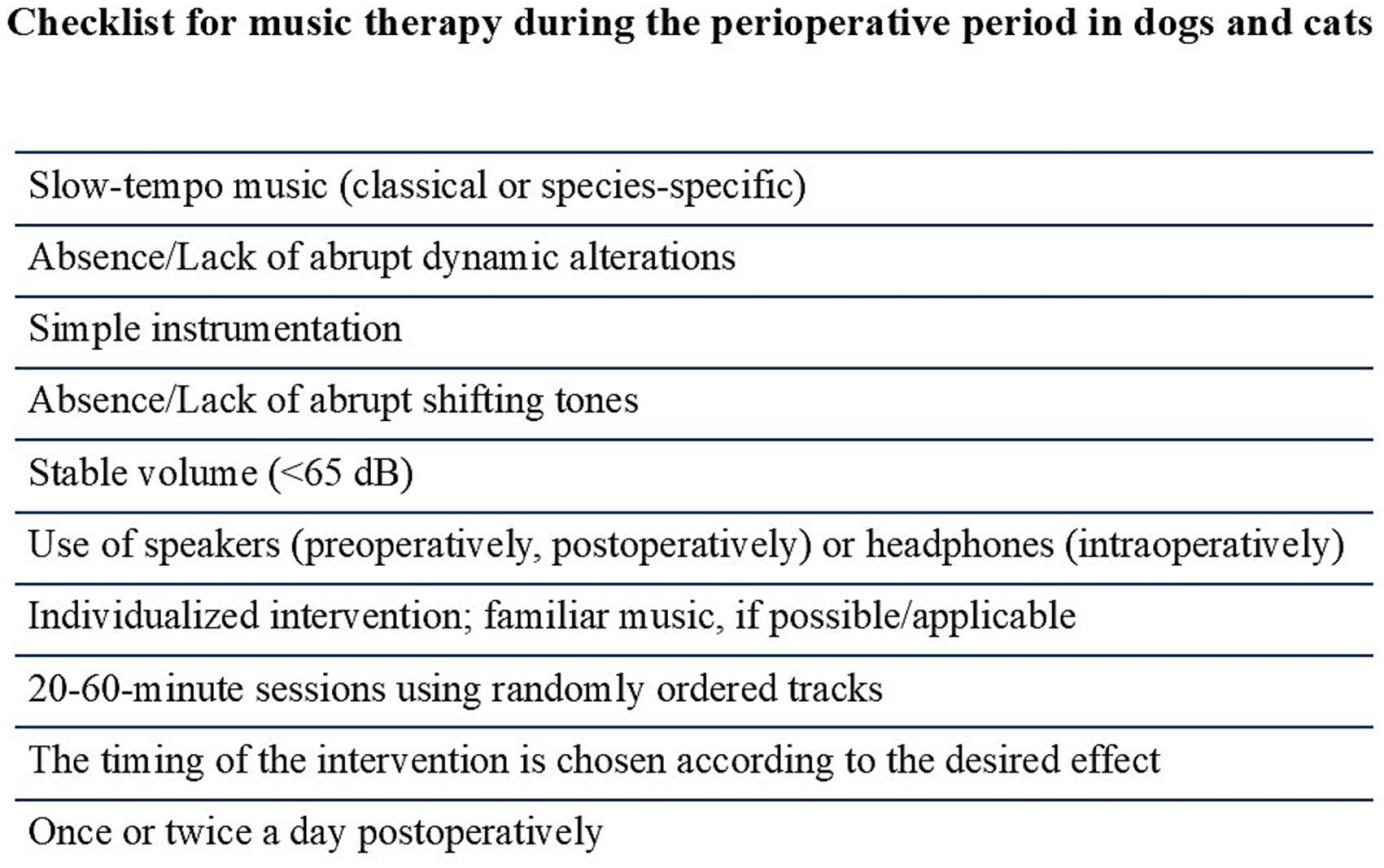
Recommendations and directions for a successful perioperative music intervention in dogs and cats.

The current review aimed to provide some recommendations and directions according to the existing literature, considering the reports on both human and domestic animals (Figure 2). By following these guidelines, music can be an effective tool to help calm dogs and cats before, during, and after surgery, improving their overall experience and potentially enhancing their recovery. The clinical implications of perioperative music are particularly relevant to veterinary anesthesiologists in practice, potentially in more challenging scenarios, beyond the already existing evidence in dogs and cats. For example, music’s anxiolytic and analgesic effects could help reduce anesthetic requirements and promote hemodynamic stability in long orthopedic surgeries. For high-risk or geriatric patients, even small reductions in sympathetic tone may decrease the need for cardiovascular support. In feline patients who are susceptible to stress-related sympathetic activation, music may facilitate smoother inductions and more stable anesthetic planes. These scenarios illustrate music’s potential to improve perioperative management and could greatly expand treatment options for veterinary patients. Music therapy should represent an adjunctive, non-pharmacological treatment option, rather than a monotherapy, in terms of a multimodal approach. In general, a therapeutic strategy involving music should not replace but rather complement the already established pharmacological interventions, providing additional anxiolysis and analgesia and optimizing the final surgical outcome. Music could be also effective when combined with other non-pharmacological strategies such as pheromone therapy or gentle handling practices to create a more holistic environment.

For a successful music intervention in dogs and cats, the environment should be quiet rather than loud and crowded, and the music should not compete against other noises. Music type is specified by the music genre, tempo, pitch and instrumentation which should be considered as a whole, as no component seems to be solely the gold standard.

- Slow-tempo classical music or music specifically designed for dogs or cats, without abrupt dynamic alterations, is recommended. Prefer simple instrumentation (e.g., piano as the sole instrument) and avoid abruptly shifting tones that could overstimulate the patient.
- Keep volume stable throughout the intervention; definitely below 85 dB, and ideally not to exceed 65 dB.
- Deliver the music with speakers during the preoperative and postoperative periods or use headphones covering the whole ear intraoperatively.
- Consider music compositions that the animal may be familiarized with. If no such information is available, follow the directions above. Firstly, acclimatize the animal to the novel environment of the preparation room, and only after that, should the music be initiated. Keep a period for the animal to be acclimatized to the auditory stimulus, as well, and do not administer the premedication until a sufficient period passes. If an aversive behavior appears, change the playlist; otherwise, wait for the maximum effect of the premedication.
- Sessions of 20–60 min should be designed; non-stop, and all-day interventions should be avoided.
- Music could be provided during the whole perioperative period, or according to the desired effect.
- Randomly order the tracks if the music intervention is meant to be used on multiple timepoints postoperatively. Do not use single-track interventions on a loop, or too short playlists, to avoid habituation.
- Music should be played once or twice a day during the postoperative period.
- The music intervention should be individualized, according to the temperament and background of the patient or according to the desired, anticipated effect.

## 4 Suggestions for potential future research

Despite the growing interest in the current research topic lately, the fact that music is a highly variable and complex intervention, and the use of heterogeneous research methods make it difficult to extrapolate definite conclusions or ensure replicability. Studies of poor methodological quality can compromise the analysis regarding the potential effects of music on dogs’ and cats’ perioperative period. The characteristics of an optimal music intervention (music genre, tempo, pitch, instrumentation, volume, method of delivery, previous music experience, duration and timing of the music intervention, and frequency of presentation) are crucial for specific therapeutic goal fulfillment and underscore the need for controlled musical interventions. Music type, for example, being the central component of the intervention, is mostly described either using vague descriptions (e.g., genre), or by employing subjective characteristics such as “relaxing music” or “soothing music.” However, describing music in such terms lacks validity and universal interpretation, supporting the need for more objective approaches when describing music according to specific musical features. On the other hand, language and concepts used by music theorists are objective, allow replication and would potentially decrease the heterogeneity of music studies. Furthermore, not all veterinary studies mention details of frequency, duration or timing of the music intervention, or rarely report information regarding previous exposure to music or individual differences in temperament of the studied populations. The timing could influence outcomes like stress reduction, anesthetic requirements or pain perception, while breed or personality differences or previous music experience could affect responsiveness to music. So, it is imperative that a music intervention should be accurately characterized and appropriately described in a research study, using all the previously mentioned components.

Another parameter that should be considered in future studies is the theoretical framework underlying the effectiveness of music. Studies should explain the reasons underlying the selection of the specific music intervention and what is expected from such an intervention. A recently published manuscript presented the potential neurobiological and physiological pathways mediating the effects of music in dogs and cats (81). According to this review, music may influence key neurophysiological mechanisms, including modulation of mesolimbic dopaminergic pathways, activation of endogenous opioid systems and attenuation of hypothalamic–pituitary–adrenal (HPA) axis activity. Music’s neurophysiological effects have been assessed in veterinary literature through biomarkers such as cortisol, as a stress-related hormone, or by HRV as an index of autonomic (sympathetic-parasympathetic) balance. So, it would probably be beneficial for future research studies to explore the potential link between music and alterations in opioid or dopamine activity and their correlation with the above objective biomarkers. However, most proposed pathways are extrapolated by human literature because of limited veterinary data, while species-specific differences such as auditory range or music preferences should be carefully considered as they may modify these responses and thus merit further investigation.

Other methodological parameters that should be controlled in future research include the employment of a proper blinding methodology (double-blind study design is recommended), adequately addressed randomization technique and power, clearly stated research questions (primary and secondary study outcomes), and well-defined means of the outcome measure evaluation. Regarding the study design, another parameter to consider is the type of the control condition, as different studies have used different control interventions; they either used total silence, typical back-ground noise depending on the environment, or other non-pharmacological interventions. In that context, comparing the results of studies using different “control” interventions may not be ideal for scientific analysis.

Finally, it would be important to clearly describe the observed effects of the musical intervention, such as autonomic alterations, stress-reducing effects, anesthetic- or analgesic-sparing effects, or any other adverse effects and complications.

Overall, it should be suggested that more RCTs need to be conducted to standardize music protocols and support clinical implementation. They should disclose detailed information about the selection of the sample, the type of the study population included (e.g., healthy vs. diseased animals, or animals with acute or pre-existing pain conditions), the exact characteristics of the musical pieces, the duration and timing of the intervention, the evaluation times, the anticipated music-induced effects by clearly stating the outcome measures and the evaluation of the potential adverse effects or postoperative complications. In terms of a more objective assessment of music’s effectiveness, future research should investigate how music affects the ANS (autonomic nervous system) and brain activity by evaluating different biomarkers (e.g., cortisol, oxytocin, dopamine, endogenous opioids); functional neuroimaging methods (e.g., fMRI, PET) or pharmacological antagonist studies could be conducted to better clarify the specific mechanism of music’s effect. By these means, the potential involvement of placebo mechanisms, or the potential activation of the reward circuitry, as has already been proposed for music interventions, could be assessed. Finally, it could be of interest to investigate whether the combined use of anxiolytic medications with music interventions or other non-pharmacological treatments could further benefit the outcomes in dogs and cats during the perioperative period.

Future research on perioperative music interventions in dogs and cats holds promising potential to enhance animal welfare by reducing stress and anxiety, minimizing the need for pharmacological agents and supporting the development of evidence-based, minimally invasive strategies to improve veterinary clinical care.
